# The effects of alpha-pinene on anxiety behaviours and TLR4/MYD88/NF-kB pathway in the hippocampus in the reserpine-induced anxiety model in rats

**DOI:** 10.1017/neu.2026.10080

**Published:** 2026-04-24

**Authors:** Matin Nasiri, Kaveh Rahimi, Zohreh Ghotbeddin

**Affiliations:** Shahid Chamran University of Ahvaz, Iran, Islamic Republic of

**Keywords:** Alpha-pinene, anxiety, inflammation, hippocampus, reserpine

## Abstract

**Objective:**

Anxiety disorders are prevalent neuropsychiatric conditions associated with neuroinflammation and altered cytokine signalling in the hippocampus. This study aimed to evaluate the anxiolytic-like effects of alpha-pinene and its potential modulation of hippocampal neuroinflammatory pathways in a reserpine-induced anxiety model.

**Methods:**

Adult male Wistar rats were randomly assigned to four groups: control (vehicle), reserpine (0.5 mg/kg, i.p.), and reserpine co-treated with alpha-pinene at 50 or 100 mg/kg. Treatments were administered intraperitoneally for 10 consecutive days. Behavioural assays – including the Open Field Test, Elevated Plus Maze, and Light/Dark Box Test – assessed locomotor activity and anxiety-like behaviours. Following testing, hippocampal tissues were collected for molecular analyses, including real-time PCR for TLR4, MyD88, and NF-κB expression, and ELISA quantification of IL-1β and IL-6 levels.

**Results:**

Reserpine induced robust anxiety-like behaviours, accompanied by significant upregulation of TLR4, MyD88, and NF-κB expression and increased hippocampal IL-1β and IL-6. Alpha-pinene treatment at both doses significantly attenuated anxiety-like behaviours and reduced neuroinflammatory markers, suggesting involvement of the TLR4/MyD88/NF-κB pathway.

**Conclusion:**

Alpha-pinene exhibits anxiolytic-like effects in reserpine-treated rats, potentially via suppression of hippocampal neuroinflammation, supporting further investigation into its therapeutic potential for anxiety disorders.

## Graphical abstract

**Figure:**
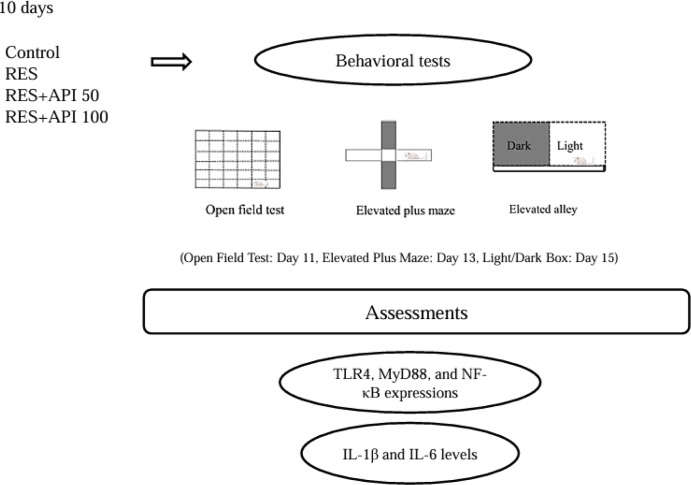

Significant outcomes

- Alpha-pinene significantly reduced reserpine-induced anxiety-like behaviours in multiple behavioural paradigms (Open Field, Elevated Plus Maze, Light/Dark Box).
- Treatment with alpha-pinene decreased hippocampal expression of TLR4, MyD88, NF-κB, and pro-inflammatory cytokines IL-1β and IL-6.
- The anxiolytic and anti-inflammatory effects of alpha-pinene are likely mediated via inhibition of the TLR4/MyD88/NF-κB signalling pathway.

Limitations

- Only male rats were used, limiting the generalizability to females or other species.
- Gene expression changes were not validated at the protein level or for functional activity.
- The study evaluated a single anxiety model (reserpine-induced) over a short 10-day period, restricting assessment of long-term or model-independent effects.

## Introduction

Anxiety is characterised by heightened vigilance and defensive reactions to potential threats, which serve an adaptive role in protecting the organism from harm (Craske & Stein, 2016). However, when neural circuits regulating mood and anxiety are disrupted by genetic or environmental factors, these adaptive responses may progress into pathological conditions such as generalised anxiety disorder, obsessive–compulsive disorder (OCD), panic disorder, post-traumatic stress disorder (PTSD), and social phobia (Craske & Stein, 2016). Pharmacological treatments such as selective serotonin reuptake inhibitors (SSRIs) are considered the first-line therapy for anxiety disorders. Additional drugs, including tricyclic antidepressants, buspirone, and pregabalin, are also prescribed. Nevertheless, many patients do not respond sufficiently, suggesting that mechanisms beyond monoamine dysfunction contribute to the pathogenesis of anxiety (Bandelow *et al*., 2017).

The hippocampus, located in the medial temporal lobe, plays a critical role not only in learning and memory but also in regulating anxiety-related behaviours (Bannerman *et al*., 2004; Revest *et al*., 2009). Activation of the inflammatory response leads to release of inflammatory cytokines which have been shown to alter behaviour (Miller *et al*., 2013). Thus, clarifying the cellular and molecular mechanisms underlying hippocampal excitability is essential for developing more effective therapies, particularly in treatment-resistant cases (Ghasemi *et al*., 2022).

Dysregulation of the immune system has also been implicated in anxiety disorders. Stress, as a key factor in anxiety, alters immune responses and increases pro-inflammatory cytokine production. Chronic stress disrupts the hypothalamic-pituitary-adrenal (HPA) axis and autonomic balance, leading to elevated inflammatory mediators that may damage neural tissue and contribute to neuroinflammation-associated anxiety (Nance & Sanders, 2007; Yehuda, 2009; Hendrickson & Raskind, 2016; Michopoulos *et al*., 2017; Patriquin & Mathew, 2017; Felger, 2018).

Reserpine, a VMAT2 inhibitor, depletes synaptic monoamines such as dopamine, norepinephrine, and serotonin, and has been widely used to model depression-like behaviours in rodents (Cheung & Parmar, 2022). Importantly, recent studies indicate that reserpine administration can also induce anxiety-like behaviours in rodents, accompanied by hippocampal neuroinflammation and increased pro-inflammatory cytokines (IL-1β, IL-6, TNF-α) (Kim *et al*., 2021; Qian *et al*., 2023; Sadeghi Nejad *et al*., 2023). Therefore, the reserpine model provides a relevant platform to study both the behavioural and molecular aspects of anxiety.

Alpha-pinene (C10H16) is a monoterpene hydrocarbon abundant in pine essential oils, present in alpha and beta isomeric forms with biologically active enantiomers. It is widely applied in food, fragrance, and pharmaceutical industries and is considered safe for use (Erman & Kane, 2008; Vespermann *et al*., 2017). Compounds containing pinene exhibit favourable safety profiles, supporting their broad chemical applications (Almirall *et al*., 1996; Vespermann, *et al*., 2017). Recent investigations have focused on the therapeutic potential of alpha-pinene, particularly its anti-inflammatory activity (Rahimi *et al*., 2023, 2025). It has been shown to reduce pain in the formalin test, suppress TNF-α and IL-1β, and decrease COX-1 expression in the spinal cord (Rahimi *et al*., 2023). Alpha-pinene improved behavioural outcomes, decreased MDA levels, and increased GPx activity in stressed rats (Hosseini, *et al*., 2025). In addition, it has recently reported that alpha-pinene improves spatial working memory in rodent models of addiction and epileptic seizure (Hashemi & Ahmadi, 2023; Ahmadi *et al*., 2026).

Based on these findings, we hypothesised that alpha-pinene could attenuate anxiety-like behaviours induced by reserpine via modulation of hippocampal neuroinflammatory pathways. The present study aimed to evaluate the anxiolytic-like effects of alpha-pinene in reserpine-treated rats and investigate its potential impact on the NF-κB/MyD88 signalling pathway in the hippocampus.

## Methods

### Animals and experimental groups

A total of 32 adult male Wistar rats (250 ± 20 g) were used. The study was approved by the Institutional Animal Care and Use Committee (IACUC approval number: IR.SCU.REC.1404.007) and conducted in accordance with the National Institutes of Health (NIH) Guide for the Care and Use of Laboratory Animals, as well as ARRIVE guidelines (Percie du Sert *et al*., 2020). Animals were housed under a 12-h light/dark cycle (lights ON from 07:00 to 19:00), at 22 ± 2°C, with ad libitum access to food and water. Behavioural experiments were performed during the light phase between 09:00 and 15:00.

### Experimental groups and vehicles

Rats were randomly assigned to four groups (*n* = 8 per group): Vehicle Control: received 1 mL/day of vehicle (0.9% saline + 1% Tween-80, i.p.) for 10 days. Reserpine: 0.5 mg/kg i.p. dissolved in the same vehicle for 10 days. Reserpine + Alpha-pinene 50 mg/kg: reserpine (0.5 mg/kg, i.p.) plus alpha-pinene 50 mg/kg i.p., administered simultaneously once daily for 10 days. Reserpine + Alpha-pinene 100 mg/kg (Hajizadeh Moghaddam *et al*., 2024): same as above but with 100 mg/kg alpha-pinene.

Alpha-pinene (% minimum by GLC 97.18, refractive index at 20°C: 1.464–1.468) was provided by Saghez Sazi Kurdistan Manufacturing Co. (Van), Iran (CAS No. 7,785-26-4, FEMA No. 2,902).

### Behavioural assessments

#### Open field test

The Open Field Test was carried out in a square plexiglass arena (50 × 50 × 50 cm) under cold white LED light at 150 lux. Each rat was placed in the centre of the arena and allowed to explore for five minutes following a five-minute habituation. Multiple behavioural parameters were recorded, including the number of rearing, grooming frequency, and total distance travelled.

#### Elevated plus maze

The Elevated Plus Maze consisted of two open arms (50 × 10 cm) and two closed arms (50 × 10 × 50 cm) elevated 50 cm above the floor. Each rat was placed at the centre facing an open arm and observed for five minutes. Time spent in the open and closed arms were recorded (Yu Ri Pellow *et al*., 1985; Kim *et al*., 2021).

#### Light/Dark box test

The apparatus included a light compartment (30 × 20 × 25 cm) and a dark compartment (30 × 20 × 25 cm), connected by a small opening. The light compartment was illuminated by cold white LED at 150 lux. Rats were placed in the light compartment and allowed to freely explore both areas for five minutes. Latency to enter the dark compartment, number of entries into the light compartment and time spent in the light compartment were measured.

Behavioural tests were performed on separate days (Open Field Test: Day 11, Elevated Plus Maze: Day 13, Light/Dark Box: Day 15) to minimise stress carryover, and all sessions were recorded for blinded analysis.

### Tissue collection

Following the behavioural tests, rats were euthanized using an overdose of ketamine-xylazine anaesthesia (100 mg/kg ketamine + 10 mg/kg xylazine, i.p.), and hippocampal tissue was quickly isolated and snap-frozen at −70°C for molecular analyses. This method ensures rapid, humane, and ethically approved termination of the animals. The left and right hippocampi in each animal were completely used for biochemical evaluation.

### Real-time PCR

Total RNA was extracted from approximately 50 mg of frozen hippocampal tissue (Day 16) using a commercial Total RNA Extraction Kit (Pars Tous, Iran) according to the manufacturer’s instructions. Samples were homogenised in liquid nitrogen, followed by chloroform extraction and ethanol precipitation. The resulting RNA pellet was washed, air-dried, and resuspended in 50 µL of RNase-free buffer. RNA concentration and purity were assessed using a NanoDrop spectrophotometer (Pishro Pazhouh, Iran) by measuring absorbance at 260 nm and calculating the 260/280 and 260/230 ratios, with acceptable thresholds set at ≥1.8 and ≥1.5, respectively. Complementary DNA (cDNA) was synthesised using the Easy cDNA Synthesis Kit (Pars Tous, Iran). The reaction mixture contained 5 µL RNA (∼1 µg), 10 µL 2X Buffer Mix (containing oligo-dT and random hexamers), 2 µL Enzyme Mix, and 3 µL nuclease-free water. The cDNA synthesis protocol involved incubation at 25°C for 10 min, 47°C for 60 min, and 85°C for 5 min. Real-time PCR was performed using a SYBR Green-based detection system (2X SYBR Green Real-Time PCR Master Mix, Pars Tous) on a RunMei Q2000 thermal cycler (RunMei, China). The expression levels of NF-κB, TLR4, and MyD88 genes were quantified relative to the housekeeping gene GAPDH using the ΔΔCt method. Primers were designed using Beacon Designer 8.1 and are listed in Table 1.

**Table 1.** Primer sequences

Gene name	Sequences	Long	No association
GAPDH -F	AGTTCAACGGCACAGTCAAG	119	NM_017008.4
GAPDH -R	TACTCAGCACCAGCATCACC		
TLR4 -F	TCAGCTTTGGTCAGTTGGCT	141	NM_019178.2
TLR4 -R	TGACACCATTGAAGCTGAGGT		
NFKB -F	TCAACATGGCAGACGACGAT	134	NM_001276711.1
NFKB -R	TTGAAGGTATGGGCCATCTGT		
MYD88 -F	GCTCATTGAGAAAAGGTGTCGT	103	NM_198130.2
MYD88 -R	AGACAGGCTGAGAGCAAACT		

### Assessment of inflammatory cytokines

To evaluate inflammatory responses, hippocampal tissue samples were first homogenised under cold conditions. Levels of pro-inflammatory IL-1β and IL-6 were quantitatively measured using enzyme-linked immunosorbent assay (ELISA) kits (Kiazist, Iran), according to the manufacturer’s protocols. All assays were performed in duplicate, and absorbance was read at 450 nm using a microplate reader (Biotech, USA). Concentrations were expressed as pg/mg total protein.

### Statistical analysis

All data were analysed using GraphPad Prism version 8. Normality of the data was assessed using the Shapiro–Wilk test. As the data met the assumptions of normal distribution, comparisons among groups were performed using one-way analysis of variance (ANOVA), followed by Tukey’s post hoc test for multiple pairwise comparisons. Results are presented as mean ± standard error of the mean (SEM), and a *p*-value < 0.05 was considered statistically significant.

## Results

### Open field test

**Figure 1.**
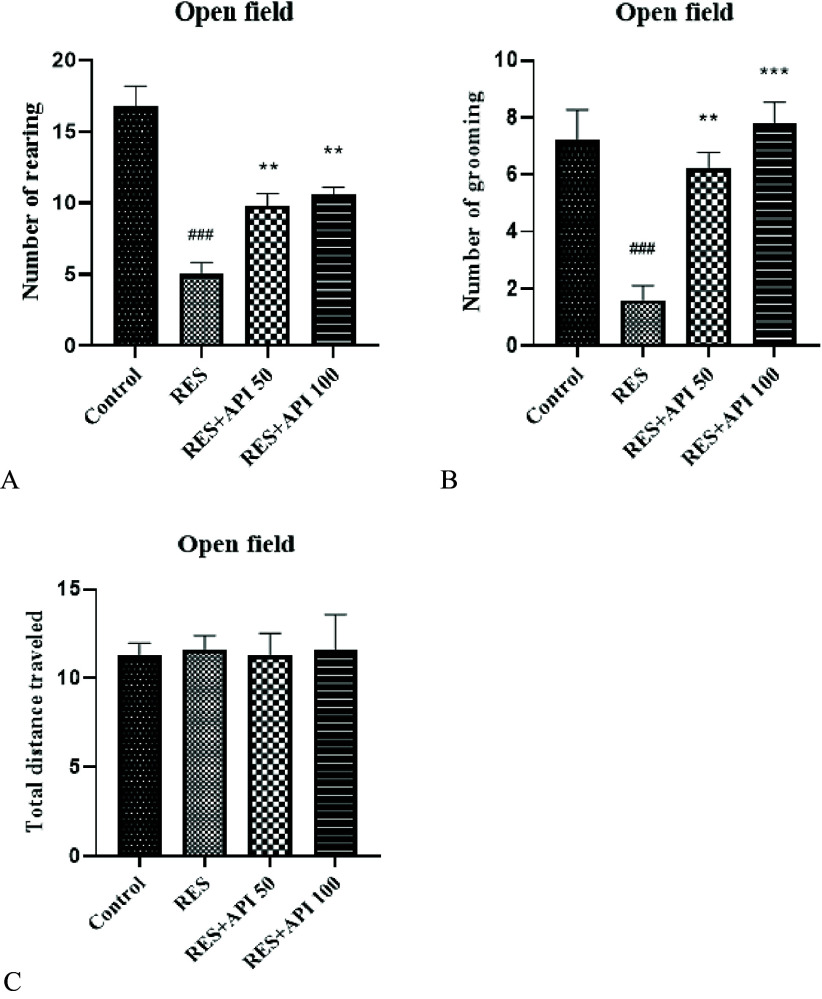
Open field test results. (A) number of rearing episodes. (B) number of grooming episodes. Data are expressed as mean ± SEM (*n* = 8). Reserpine group (RES), reserpine + alpha-pinene 50 (RES + API 50) and reserpine + alpha-pinene 100 (RES + API 100). #*P* < 0.05, ##*P* < 0.01, ###*P* < 0.001 indicate comparison between the RES group and the control group. *P < 0.05, **P < 0.01, ***P < 0.001 indicate comparison between the RES + API 50/100 groups and the RES group.

In the Open Field Test, one-way ANOVA revealed a significant effect of treatment on the number of rearing episodes F(3,16) = 25.80, P < 0.0001, R^2^ = 0.83. Post hoc analysis showed that the number of rearing in the Reserpine group (5.00 ± 0.83) was significantly lower than in the Control group (16.80 ± 1.39, *P* < 0.001). Treatment with Alpha-Pinene increased rearing behaviour, with both Reserpine + Alpha-Pinene 50 (9.80 ± 0.86) and Reserpine + Alpha-Pinene 100 (10.60 ± 0.50) showing significant improvements relative to Reserpine (*P* = 0.01 and *P* = 0.004, respectively). No significant difference was observed between the two Alpha-Pinene doses (Figure 1A).

Similarly, one-way ANOVA revealed a significant effect of treatment on the number of grooming episodes in the Open Field Test F(3,16) = 13.87, P = 0.0001, R^2^ = 0.72. The assumption of homogeneity of variances was satisfied, as indicated by non-significant Brown–Forsythe (*P* = 0.79) and Bartlett’s tests (*P* = 0.49). Post hoc analysis demonstrated that grooming behaviour was significantly reduced in the Reserpine group (1.60 ± 0.50) compared with the Control group (7.20 ± 1.06, *P* = 0.0004). Both Alpha-Pinene treatments significantly increased the number of grooming episodes relative to Reserpine-treated animals, with Reserpine + Alpha-Pinene 50 (6.20 ± 0.58, *P* = 0.003) and Reserpine + Alpha-Pinene 100 (7.80 ± 0.73, *P* = 0.0001). No significant difference was observed between the two Alpha-Pinene doses (Figure 1B).

The total distance travelled in the Open Field Test did not differ significantly among groups (Control group: 11.27 ± 0.32, Reserpine group: 11.60 ± 0.36, Reserpine + Alpha-Pinene 50: 11.29 ± 0.56, Reserpine + Alpha-Pinene 100: 11.62 ± 0.88; one-way ANOVA, *F*(3,16) = 0.109, *P* = 0.954). Homogeneity of variances was confirmed by Brown–Forsythe (*P* = 0.245) and Bartlett’s test (*P* = 0.195) (Figure 1C).

### Elevated plus maze

**Figure 2.**
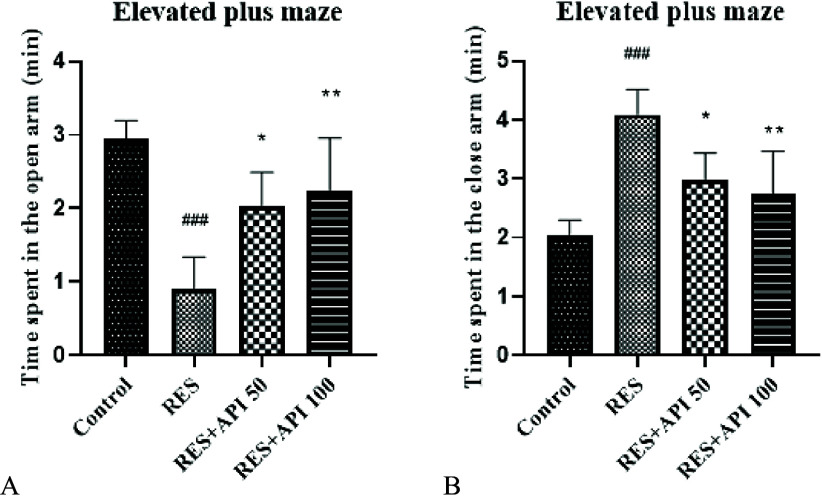
Elevated plus maze results. (A) time spent in the open arms. (B) time spent in the closed arms. Data are expressed as mean ± SEM (*n* = 8). Reserpine group (RES), reserpine + alpha-pinene 50 (RES + API 50) and reserpine + alpha-pinene 100 (RES + API 100). #*P* < 0.05, ##*P* < 0.01, ###*P* < 0.001 vs. control group; *P < 0.05, **P < 0.01, ***P < 0.001 vs. RES group.

In the Elevated Plus Maze, one-way ANOVA revealed a significant effect of treatment on the time spent in the open arms F(3,16) = 11.84, P = 0.0002, R^2^ = 0.69. The assumption of homogeneity of variances was satisfied, as confirmed by non-significant Brown–Forsythe (*P* = 0.69) and Bartlett’s tests (*P* = 0.77). Post hoc analysis showed that time spent in the open arms was significantly reduced in the Reserpine group (0.91 ± 0.19 s) compared with the Control group (2.95 ± 0.25 s, *P* = 0.0001). Treatment with Alpha-Pinene at both doses significantly increased open-arm exploration relative to Reserpine-treated animals, with Alpha-Pinene 50 (2.03 ± 0.21 s, *P* = 0.025) and Alpha-Pinene 100 (2.25 ± 0.32 s, *P* = 0.007), indicating a moderate anxiolytic-like effect. No significant difference was observed between the two Alpha-Pinene doses (Figure 2A).

In the Elevated Plus Maze, one-way ANOVA revealed a significant effect of treatment on the time spent in the closed arms F(3,16) = 11.84, P = 0.0002, R^2^ = 0.69. The assumption of homogeneity of variances was met, as indicated by non-significant Brown–Forsythe (*P* = 0.69) and Bartlett’s tests (*P* = 0.77). Post hoc analysis demonstrated that time spent in the closed arms was significantly increased in the Reserpine group (4.09 ± 0.19 s) compared with the Control group (2.05 ± 0.25 s, *P* = 0.0001). Both Alpha-Pinene treatments significantly reduced closed-arm occupancy relative to Reserpine-treated animals, with Alpha-Pinene 50 (2.97 ± 0.21 s, *P* = 0.025) and Alpha-Pinene 100 (2.75 ± 0.32 s, *P* = 0.007). No significant difference was observed between the two Alpha-Pinene doses (Figure 2B).

### Light/Dark box test

**Figure 3.**
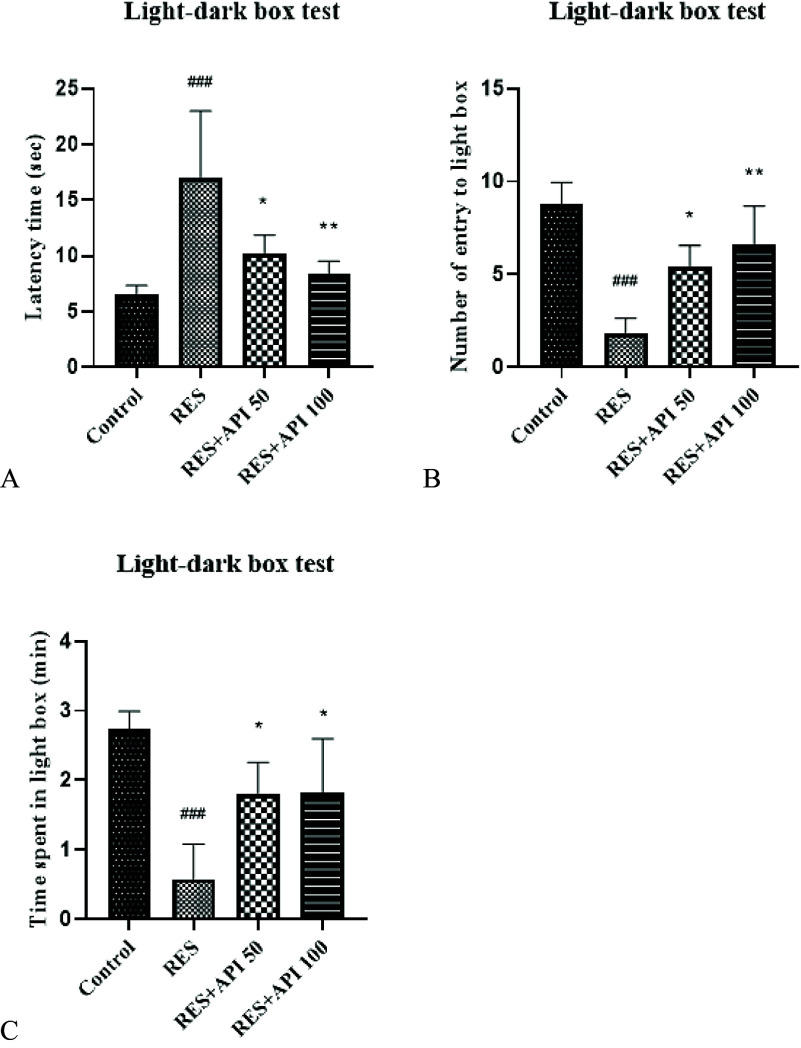
Light/dark box results. (A) latency to enter the dark compartment. (B) number of entries into the light compartment. (C) time spent in the light compartment. Data are expressed as mean ± SEM (*n* = 8). Reserpine group (RES), reserpine + alpha-pinene 50 (RES + API 50) and reserpine + alpha-pinene 100 (RES + API 100). #*P* < 0.05, ##*P* < 0.01, ###*P* < 0.001 vs. control group; *P < 0.05, **P < 0.01, ***P < 0.001 vs. RES group.

In the Light–Dark Box test, one-way ANOVA revealed a significant effect of treatment on latency to enter the dark compartment *F*(3,16) = 9.65, *P* = 0.0007, *R* ^2^ = 0.64. Homogeneity of variances was partially met: the Brown–Forsythe test was not significant (*P* = 0.36), while Bartlett’s test indicated a significant difference among variances (*P* = 0.0045). Post hoc analysis showed that Reserpine treatment significantly increased latency to enter the dark compartment (17.00 ± 2.68 s) compared with the Control group (6.60 ± 0.75 s, *P* = 0.0006), suggesting heightened anxiety-like behaviour. Both Alpha-Pinene treatments attenuated this effect, with Alpha-Pinene 50 (10.20 ± 0.74 s, *P* = 0.022) and Alpha-Pinene 100 (8.40 ± 0.51 s, *P* = 0.004) significantly reducing latency relative to the Reserpine group. No significant difference was observed between the two Alpha-Pinene doses (Figure 3A).

In the Light–Dark Box test, one-way ANOVA revealed a significant effect of treatment on the number of entries into the light compartment *F*(3,16) = 13.18, *P* = 0.0001, *R* ^2^ = 0.71. Assumptions of homogeneity of variances were met, as indicated by non-significant Brown–Forsythe (*P* = 0.18) and Bartlett’s tests (*P* = 0.15). Post hoc comparisons showed that Reserpine-treated animals exhibited a significant reduction in light compartment entries (1.80 ± 0.37) compared with the Control group (8.80 ± 1.16, *P* < 0.0001), reflecting increased anxiety-like behaviour. Both doses of Alpha-Pinene significantly reversed this effect, with Alpha-Pinene 50 (5.40 ± 0.51, *P* = 0.028) and Alpha-Pinene 100 (6.60 ± 0.93, *P* = 0.003) groups showing higher light compartment entries relative to Reserpine. No significant difference was observed between the two Alpha-Pinene doses (Figure 3B).

In the Light–Dark Box test, one-way ANOVA revealed a significant effect of treatment on the time spent in the light compartment *F*(3,16) = 11.19, *P* = 0.0003, *R* ^2^ = 0.68. Assumptions of homogeneity of variances were met, as indicated by non-significant Brown–Forsythe (*P* = 0.995) and Bartlett’s tests (*P* = 0.73). Post hoc analysis demonstrated that Reserpine treatment significantly reduced time spent in the light compartment (0.58 ± 0.23 s) compared with the Control group (2.73 ± 0.26 s, *P* = 0.0002), consistent with increased anxiety-like behaviour. Both Alpha-Pinene treatments partially reversed this effect, with Alpha-Pinene 50 (1.80 ± 0.20 s, *P* = 0.022) and Alpha-Pinene 100 (1.82 ± 0.35 s, *P* = 0.021) showing significant increases in light compartment duration relative to Reserpine. No significant difference was observed between the two Alpha-Pinene doses (Figure 3C).

### Gene expression

**Figure 4.**
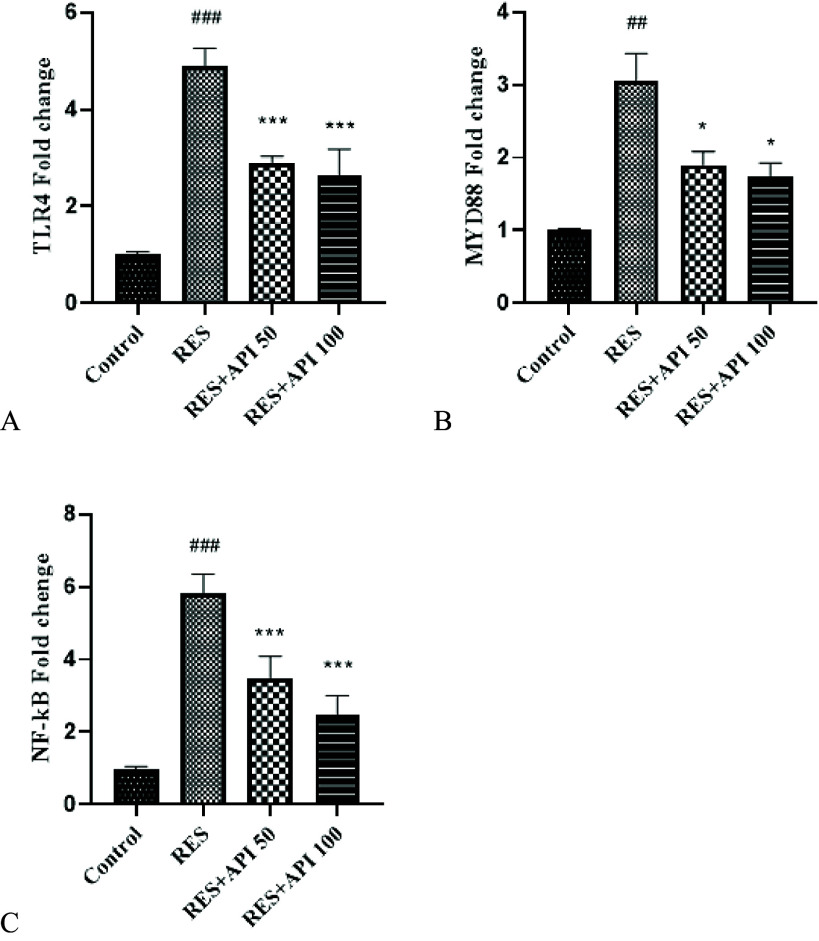
Gene expression results. (A) TLR4, (B) MYD88, (C) NF-κB. Data are expressed as mean ± SEM (*n* = 8). Reserpine group (RES), reserpine + alpha-pinene 50 (RES + API 50) and reserpine + alpha-pinene 100 (RES + API 100). #*P* < 0.05, ##*P* < 0.01, ###*P* < 0.001 vs. control group; *P < 0.05, **P < 0.01, ***P < 0.001 vs. RES group.

Analysis of TLR4 expression revealed a significant effect of treatment *F*(3,12) = 89.23, *P* < 0.0001, *R* ^2^ = 0.96. Variance homogeneity was largely satisfied, as indicated by a non-significant Brown–Forsythe test (*P* = 0.17), although Bartlett’s test suggested some difference among variances (*P* = 0.014). Post hoc comparisons showed that Reserpine treatment significantly increased TLR4 expression (4.90 ± 0.18) compared with the Control group (0.99 ± 0.03, *P* < 0.0001). Both Alpha-Pinene treatments markedly reduced TLR4 levels relative to Reserpine, with Alpha-Pinene 50 (2.90 ± 0.07) and Alpha-Pinene 100 (2.63 ± 0.28, *P* < 0.0001), indicating a dose-dependent attenuation of Reserpine-induced upregulation. No significant difference was observed between the two Alpha-Pinene doses (Figure 4A).

Analysis of MYD88 expression revealed a significant effect of treatment *F*(3,4) = 25.85, *P* = 0.0044, *R* **^2^** = 0.95. Homogeneity of variances was not fully met, as indicated by a significant Brown–Forsythe test (*P* < 0.0001), suggesting heterogeneity among groups. Post hoc comparisons showed that Reserpine treatment significantly elevated MYD88 expression (3.05 ± 0.27) relative to the Control group (1.01 ± 0.02, *P* = 0.003). Both Alpha-Pinene treatments significantly attenuated this upregulation, with Alpha-Pinene 50 (1.90 ± 0.14, *P* = 0.027) and Alpha-Pinene 100 (1.73 ± 0.14, *P* = 0.017). No significant difference was observed between the two Alpha-Pinene doses (Figure 4B).

Analysis of NF-κB expression revealed a significant effect of treatment *F*(3,12) = 68.70, *P* < 0.0001, *R* ^2^ = 0.95. Variance homogeneity was mostly satisfied, as indicated by a non-significant Brown–Forsythe test (*P* = 0.36), although Bartlett’s test suggested some difference among variances (*P* = 0.034). Post hoc analysis demonstrated that Reserpine treatment significantly increased NF-κB expression (5.80 ± 0.27) compared with the Control group (0.99 ± 0.03, *P* < 0.0001). Both Alpha-Pinene treatments significantly reduced NF-κB levels relative to Reserpine, with Alpha-Pinene 50 (3.48 ± 0.31, *P* = 0.0001) and Alpha-Pinene 100 (2.48 ± 0.26, *P* < 0.0001), indicating a dose-dependent suppression. No significant difference was observed between the two Alpha-Pinene doses (Figure 4C).

### Biochemical parameters

**Figure 5.**
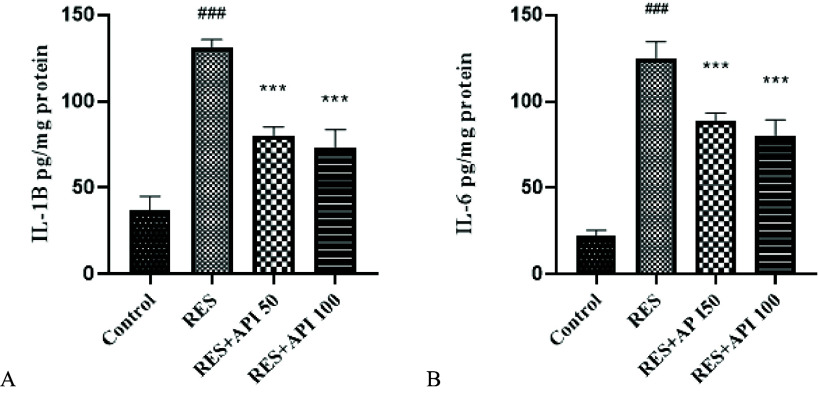
ELISA results. (A) IL-1β levels. (B) IL-6 levels. Data are expressed as mean ± SEM (*n* = 8). Reserpine group (RES), reserpine + alpha-pinene 50 (RES + API 50) and reserpine + alpha-pinene 100 (RES + API 100). #*P* < 0.05, ##*P* < 0.01, ###*P* < 0.001 vs. control group; *P < 0.05, **P < 0.01, ***P < 0.001 vs. RES group.

Analysis of IL-1β levels revealed a significant effect of treatment *F*(3,12) = 111.5, *P* < 0.0001, *R* ^2^ = 0.97. Assumptions of homogeneity of variances were met, as indicated by non-significant Brown–Forsythe (*P* = 0.77) and Bartlett’s tests (*P* = 0.51). Post hoc analysis demonstrated that Reserpine treatment significantly elevated IL-1β levels (131.0 ± 2.39 pg/mL) compared with the Control group (37.29 ± 3.87 pg/mL, *P* < 0.0001). Both Alpha-Pinene treatments significantly reduced IL-1β concentrations relative to Reserpine, with Alpha-Pinene 50 (80.34 ± 2.41 pg/mL, *P* < 0.0001) and Alpha-Pinene 100 (95.08 ± 5.21 pg/mL, *P* < 0.0001). No significant difference was observed between the two Alpha-Pinene doses (Figure 5A).

Analysis of IL-6 levels revealed a significant effect of treatment *F*(3,12) = 134.4, *P* < 0.0001, *R* ^2^ = 0.97. Homogeneity of variances was satisfied, as indicated by non-significant Brown–Forsythe (*P* = 0.28) and Bartlett’s tests (*P* = 0.22). Post hoc comparisons showed that Reserpine treatment significantly elevated IL-6 levels (124.7 ± 4.90 pg/mL) relative to the Control group (22.32 ± 1.56 pg/mL, *P* < 0.0001). Both Alpha-Pinene treatments significantly attenuated this increase, with Alpha-Pinene 50 (88.86 ± 2.15 pg/mL, *P* < 0.0001) and Alpha-Pinene 100 (79.97 ± 4.74 pg/mL, *P* < 0.0001). No significant difference was observed between the two Alpha-Pinene doses (Figure 5B).

## Discussion

The present study investigated the effects of alpha-pinene on anxiety-like behaviours and neuroinflammatory signalling in a reserpine-induced model of anxiety in rats. The main findings demonstrate that reserpine induces pronounced anxiety-like behaviours accompanied by activation of the hippocampal TLR4/MYD88/NF-κB signalling pathway and increased levels of pro-inflammatory cytokines IL-1β and IL-6. Importantly, treatment with alpha-pinene effectively attenuated both behavioural and molecular alterations, suggesting a close association between its anxiolytic effects and modulation of neuroinflammatory processes.

In the present study, reserpine administration produced robust anxiety-like behaviours in the open field, elevated plus maze, and light/dark box tests. These findings are consistent with previous studies demonstrating that reserpine disrupts monoaminergic transmission and induces anxiety- and depression-like phenotypes in rodents (Kim *et al*., 2021; Cheung & Parmar, 2022; Qian *et al*., 2023). The consistency of anxiety-like behaviours across multiple paradigms strengthens the validity of the model used in this study.

Treatment with alpha-pinene at both tested doses significantly ameliorated reserpine-induced behavioural deficits. This finding aligns with earlier reports showing beneficial behavioural effects of alpha-pinene in models of stress, hypoxia, schizophrenia, and neurotoxicity (Bakhtazad *et al*., 2024; Hajizadeh Moghaddam *et al*., 2024; Hosseini *et al*., 2025; Ahmadi *et al*., 2026). Our results extend these observations by demonstrating a clear anxiolytic-like effect of alpha-pinene in a reserpine-induced anxiety model, supporting its potential role as a novel anxiolytic compound.

At the molecular level, reserpine significantly increased the hippocampal expression of TLR4, MYD88, and NF-κB genes. Activation of this pathway has been widely implicated in stress-related neuroinflammation and emotional disorders (Gárate *et al*., 2013; Liu *et al*., 2014; García-Bueno *et al*., 2016). In line with these studies, our findings suggest that reserpine-induced anxiety is closely associated with enhanced innate immune signalling in the hippocampus.

Alpha-pinene treatment markedly suppressed the expression of TLR4, MYD88, and NF-κB. This result is particularly important, as pharmacological inhibition of TLR4 signalling has been shown to reduce stress-induced neuroinflammation and behavioural disturbances (Gárate *et al*., 2013). While previous studies have reported anti-inflammatory effects of alpha-pinene, the present study is among the first to demonstrate its regulatory effect on the TLR4/MYD88/NF-κB pathway in an anxiety-related context.

Consistent with activation of NF-κB signalling, reserpine significantly increased hippocampal levels of IL-1β and IL-6. These cytokines are known to interfere with synaptic plasticity, hippocampal function, and emotional regulation, and their elevation has been associated with anxiety and mood disorders (Miller *et al*., 2013; Michopoulos *et al*., 2017; Felger 2018). Our findings support the notion that hippocampal cytokine dysregulation contributes to the behavioural manifestations of anxiety.

Alpha-pinene significantly reduced IL-1β and IL-6 levels, indicating effective suppression of neuroinflammatory responses. Similar cytokine-lowering effects of alpha-pinene have been reported (Bakhtazad *et al*., 2024; Hosseini *et al*., 2025). The parallel improvement in behavioural outcomes and cytokine levels observed in the present study suggests a mechanistic link between reduced neuroinflammation and anxiolytic effects.

Current pharmacological treatments for anxiety disorders primarily target neurotransmitter systems and are often limited by side effects and incomplete efficacy (Bandelow *et al*., 2017). Increasing evidence suggests that neuroinflammation is a key contributor to anxiety pathophysiology (Michopoulos *et al*., 2017; Patriquin & Mathew, 2017). The present study highlights the TLR4/MYD88/NF-κB pathway as a critical mediator of anxiety-like behaviour and identifies alpha-pinene as a natural compound capable of modulating this pathway.

Importantly, this study provides novel evidence linking alpha-pinene’s anxiolytic effects to suppression of hippocampal innate immune signalling in a reserpine-induced anxiety model. These findings support the concept that targeting neuroimmune mechanisms may represent a promising strategy for the development of next-generation anxiolytic therapies.

While the present study provides robust behavioural and molecular evidence for the anxiolytic effects of alpha-pinene, future studies may further extend these findings by including female subjects, complementary protein-level analyses, additional anxiety models, and longer treatment durations to enhance translational relevance.

## Conclusion

In conclusion, the present study demonstrates that reserpine-induced anxiety-like behaviour is closely associated with activation of the hippocampal TLR4/MYD88/NF-κB pathway and increased pro-inflammatory cytokine levels. Alpha-pinene effectively attenuated both behavioural and molecular alterations, supporting its potential as a novel anxiolytic agent that targets neuroinflammatory mechanisms. These findings underscore the importance of hippocampal immune signalling in anxiety disorders and provide a foundation for future translational studies.

## Data Availability

All data generated or analysed during this study are included in this published article.
